# Disseminated Histoplasmosis in an Adult With Rheumatoid Arthritis Not on Biological Immune Modulators

**DOI:** 10.7759/cureus.15709

**Published:** 2021-06-17

**Authors:** Deepti Avasthi, Huda Fatima, Mohinder Gill, Salil Avasthi

**Affiliations:** 1 Internal Medicine, St. Vincent Mercy Medical Center, Toledo, USA; 2 Pulmonary Critical Care, St. Vincent Mercy Medical Center, Toledo, USA

**Keywords:** histoplasmosis, rheumatoid, tnf inhibitors, oral mass, cns histoplasmosis

## Abstract

Histoplasmosis is a fungal disease caused by a dimorphic fungus known as *Histoplasma capsulatum (H. capsulatum),* which is endemic to areas around river valleys and southeastern states in the United States (US). Patients with histoplasmosis are asymptomatic, and the condition is usually diagnosed by an incidental finding of a pulmonary granuloma on a chest radiograph. In rare cases, this disease can develop into a progressive disseminated form and cause fatal and diffuse pulmonary infiltrates in immunocompromised adults. Moreover, there is a close association between disseminated histoplasmosis and the use of tumor necrosis factor (TNF) inhibitors in rheumatoid arthritis (RA). Our case report discusses a unique presentation of disseminated histoplasmosis in a patient with RA who was not on any biological immune modulators. The disseminated histoplasmosis in this case was progressive and involved the central nervous system, liver, lungs, and oral mucosa and was treated successfully with amphotericin therapy. We also discuss the disease process in detail and hypothesize that RA could be an independent risk factor for the increased incidence of disseminated histoplasmosis in adults. Based on the findings in this case report, we recommend screening for latent *Histoplasma* infections in adults with RA living in endemic areas and keeping a low threshold to evaluate flare-ups from this disease regardless of the use of anti-TNF inhibitors. Specific experimental and epidemiological studies can be conducted to examine the association between RA and similar indolent fungal infections.

## Introduction

The disseminated form of histoplasmosis is the most fatal form of chronic histoplasmosis and is associated with 100% mortality if left untreated. It frequently affects immunocompromised patients, with most cases reported among HIV-infected subjects and subjects using tumor necrosis factor (TNF) inhibitors. In rare cases, disseminated histoplasmosis can also occur in immunocompetent adults. The reason behind this finding is not well established but some studies attribute it to a defect in T cell immunity. In this report, we discuss a rare presentation of disseminated histoplasmosis in a patient with rheumatoid arthritis (RA) who was not on TNF inhibitors.

## Case presentation

A 61-year-old man presented to the emergency department of our hospital complaining of painful swelling on the left side of his mouth and progressive respiratory distress. As per the patient, he had noticed a sore on the gums in his left lower jaw a few weeks earlier, which had progressively enlarged and become severely painful. It was associated with hoarseness of voice and difficulty in chewing and articulation of speech. His symptoms were associated with low-grade fever and subjective chills. The patient also reported progressive dyspnea with intermittent wheezing and severe fatigue.

His medical history was significant for coronary artery disease requiring coronary angioplasty with a drug-eluting stent three years ago, atrial fibrillation for three years, diabetes mellitus type 2 for five years, RA for two years, and a foot drop for one month. He was not on any disease-modifying anti-rheumatic drugs (DMARDS). He had taken prednisone 5 mg orally once a day for one year but had recently increased the dose to 10 mg twice a day a month prior to the presentation. The DMARDS that the patient had tried in the past were tofacitinib (Janus kinase inhibitor), which had been stopped five months ago, and infliximab (TNF inhibitor) that had been stopped 10 months ago. He denied any use of methotrexate or leflunomide in the past year due to recurrent soft-tissue infections from methicillin-resistant *Staphylococcus aureus* while on these medications. The additional chronic medications taken by the patient were metformin, apixaban, aspirin, clopidogrel, and atorvastatin.

**Figure 1:**
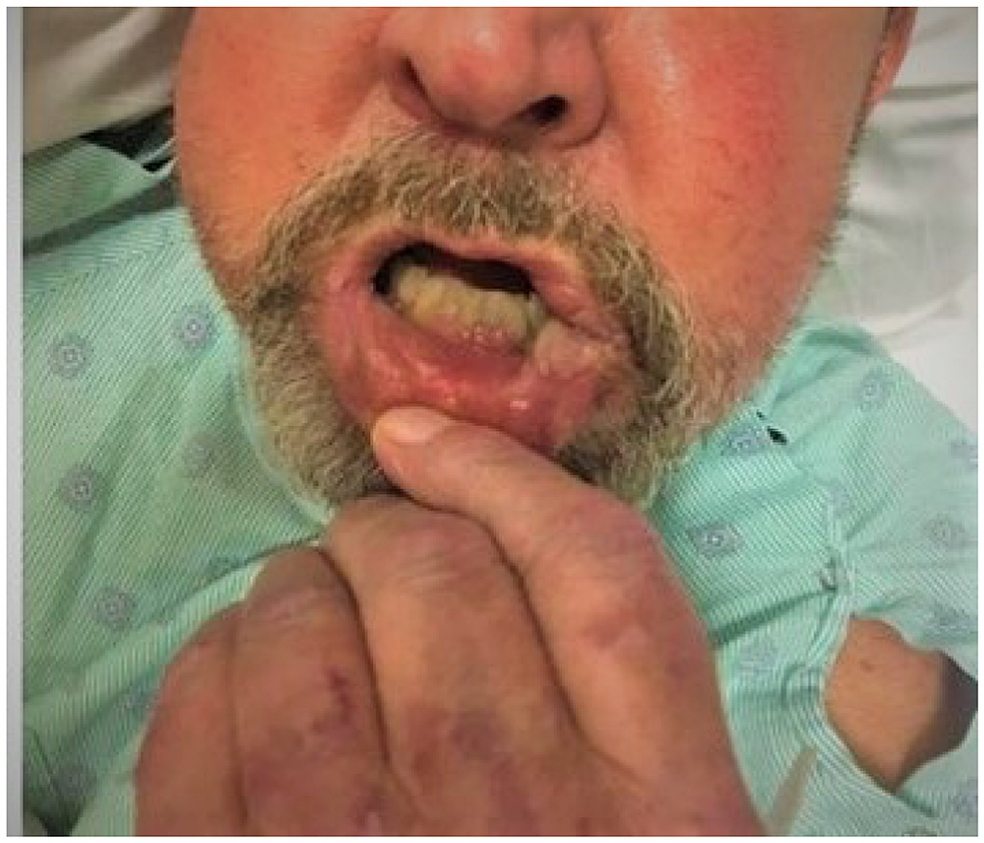
Image showing oral manifestation of disseminated histoplasmosis as a fungating mass

**Figure 2:**
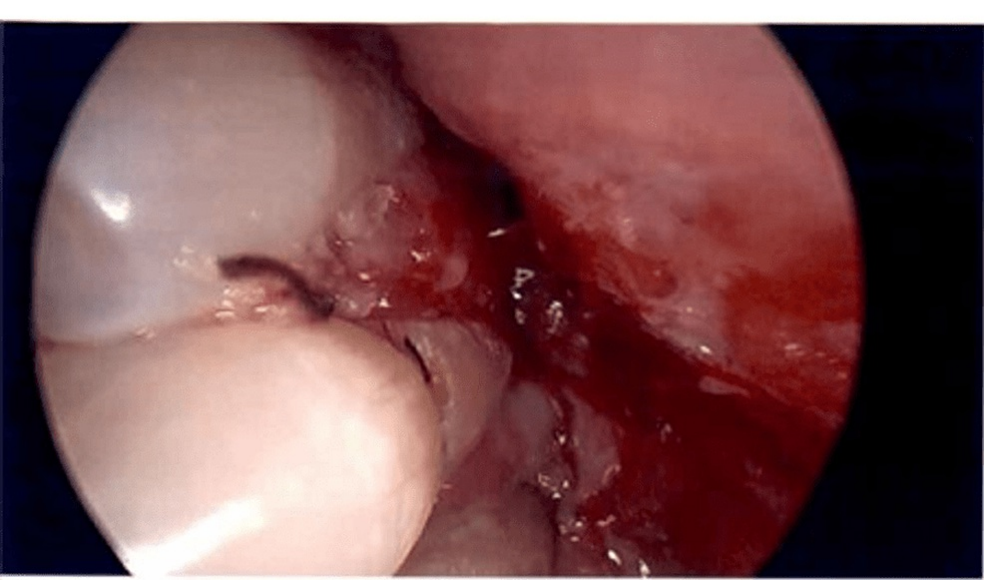
The oral examination of the patient showed an excoriating ulcer of disseminated histoplasmosis

On examination, the patient had a large fungating lesion (3 x 5 cm in size) on the left gingivobuccal sulcus causing trismus, an edematous lesion on the buccal side of the lower lip with ulceration of the commissure, tobacco-stained dentition with exposed roots, and a crusted and excoriating ulcer on the right anterior septum and nasal floor (Figures 1, 2).

The lesion was suggestive of oral malignancy. Lung auscultation revealed bilateral wheezes on the lower lung fields. Musculoskeletal examination revealed infected ulceration on the left thumb and volar aspect of the left palm and nodules on the proximal interphalangeal joints and on the dorsal aspect of both arms. On neurological examination, the patient was alert and oriented to time, place, and people with a normal cranial nerve examination and strength in his upper limbs and left leg. We noted the presence of a right foot drop and point tenderness in the lumbar spine at L2. The lab investigations of the patient, including the metabolic panel and immune panel, are listed in Table 1 and Table 2.

**Table 1:** Basic metabolic panel

Sodium (normal range: 135-145 mmol/l)	Potassium (normal range: 3.5-5 mmol/l)	Chloride (normal range: 98-107 mmol/l)	Bicarbonate (normal range: 20-28 mmol/l)	Creatinine (normal range: 0.7-1.2 mmol/l)	Lactate (normal range: 0.7-2 mmol/l)	Alkaline phosphatase (normal range: 40-129 u/l)
140	4.1	103	23	1.54	2.6	131

**Table 2:** Immune panel WBC: white blood cells; Hb: hemoglobin; ANA: antinuclear antibody; ANCA: antineutrophil cytoplasmic antibodies; MPO: myeloperoxidase; PR3: proteinase 3; CCP: cyclic citrullinated peptide

WBC (normal range: 3.5-11.3 k/ul)	Hb (normal range: 13-17 g/dl)	ANA (normal reference: negative)	ANCA-MPO (normal reference: <100 AU/ml)	ANCA-PR3 (normal reference: <100 AU/ml)	CCP IGA Ab (normal reference: <4.0 u/ml)
10.9	12.2	Negative	8	3	>200

Blood cultures were collected on July 26, 2020, and reported no growth for six days.

**Figure 3:**
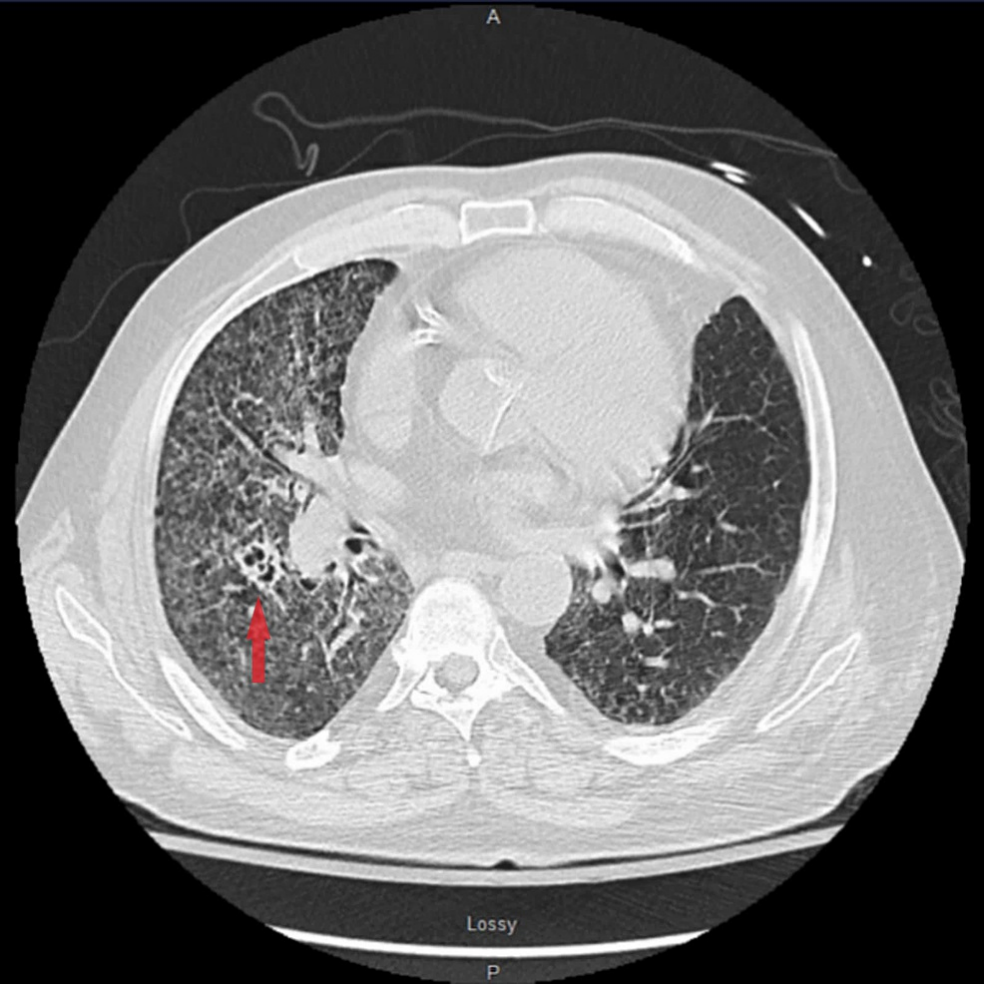
CT lungs showing diffuse micronodular changes in right lung in disseminated histoplasmosis (arrow) CT: computed tomography

**Figure 4:**
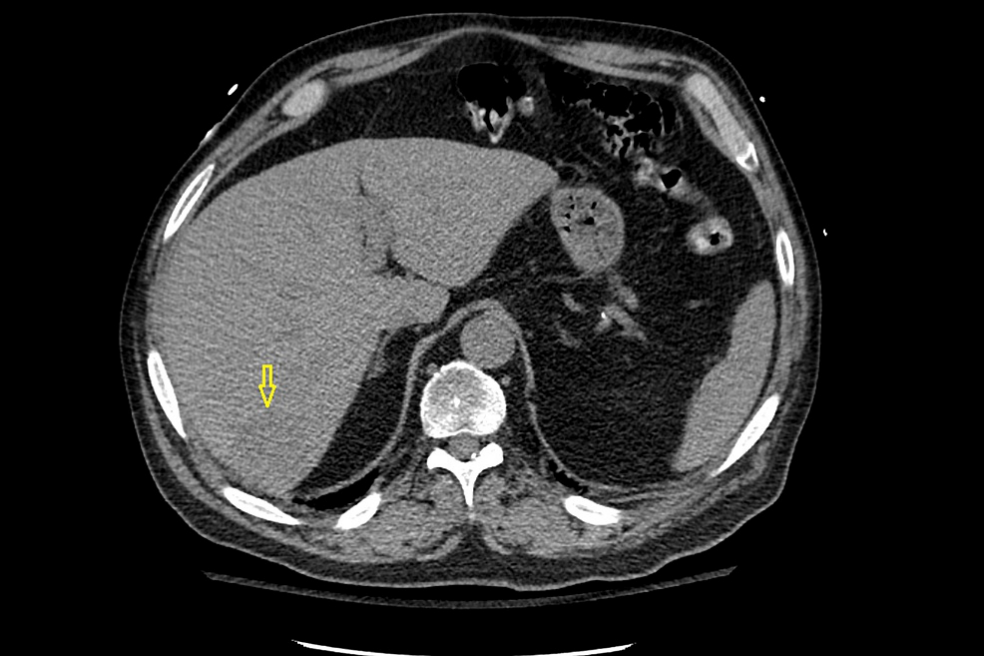
CT liver showing nodule in disseminated histoplasmosis (arrow) CT: computed tomography

The patient was admitted to the intensive care unit due to progressive respiratory distress. CT of the chest with contrast revealed multiple right perihilar irregular cavitary nodules and right-sided bronchiectasis with bilateral diffuse ground-glass attenuation, interstitial thickening, and micronodularity, more in the right lung than the left (Figure 3). No lymphadenopathy was noted. An incidental 4-cm hypoattenuating mass in the posterior right hepatic lobe was noted (Figure 4). The mass in the right hepatic lobe, the pulmonary findings, and the foot drop indicated metastatic spread throughout the body from the oropharyngeal mass. However, since an underlying septic process was not yet ruled out, empirical treatment with intravenous aztreonam and clindamycin was initiated while the confirmation of the diagnosis was underway.

MRI of the lumbar spine revealed a subacute fracture at the L2 vertebra and was negative for any mass, granuloma, or metastatic lesion.

The biopsy of the oral mass was performed via direct laryngoscopy and was negative for malignancy. The histology of the mass showed necrotizing granulomatous inflammation. The mucosa showed prominent chronic inflammation, mostly plasma cells with fewer intermingled lymphocytes and monocytes and foci of granuloma. Grocott staining and Gomori’s methenamine silver staining were positive for narrow-based budding yeast within areas of granulomatous inflammation and necrosis. Staining for acid-fast bacilli was negative; treponema immunostaining was also negative, and no viral inclusions were detected. Gram staining was negative for bacterial forms associated with granulomas. These findings were consistent with *Histoplasma* infection. The pathology findings were confirmed by a second set of pathologists at a different facility.

The patient was diagnosed with disseminated histoplasmosis with the involvement of the lung, liver, and oral cavity based on the clinical presentation and pathology confirmation. He was started on itraconazole, and treatment with intravenous antibiotics was discontinued.

After 13 days of hospital stay, the patient`s condition improved, and he was discharged to a nursing care facility close to his family in a different town. However, a few days later, his health deteriorated as he developed altered mentation and confusion. This time, the patient was admitted to a different tertiary hospital for the management of his symptoms. MRI of the brain revealed new brain lesions suggestive of the spread of disseminated histoplasmosis to the central nervous system. He was subsequently treated with amphotericin B infusions and discharged once his condition improved.

Presently (at 10-month follow-up since discharge), the patient has been noted to have achieved complete recovery from disseminated histoplasmosis of the lung and is not on oxygen supplementation anymore. His oral, liver, and brain lesions have completely resolved. He is being maintained on chronic suppressive therapy with itraconazole to prevent disease recurrence. His health is debilitated due to uncontrolled rheumatoid disease. He underwent a total knee arthroplasty during this period with a prosthetic knee implant, which was later removed following the development of a septic joint. He remains apprehensive of a possible flare-up of his histoplasmosis and secondary infections and refuses to try any immune-modulatory drugs for his RA.

## Discussion

*Histoplasma capsulatum (H. capsulatum)* is a dimorphic fungus and grows in hyphal forms in soil and bird and bat guano. When it is disturbed, the conidia become airborne and can be inhaled. It is endemic to areas around river valleys and southeastern states in the United States (US). Additional endemic foci are observed in China, Southeast Asia, Australia, and Africa [1]. Acute histoplasmosis is characterized by a self-limiting pulmonary infection that causes symptoms similar to that of influenza. Chronic histoplasmosis may appear clinically similar to tuberculosis, primarily affecting the lungs. The disseminated form of histoplasmosis frequently affects immunocompromised individuals, especially HIV patients and those using TNF inhibitors. This is the most fatal form of the disease and may affect multiple organ systems such as the spleen, adrenal glands, liver, lymph nodes, gastrointestinal tract, central nervous system, kidneys, and oral mucosa. The mortality rate is 100% without treatment. Although rare, disseminated histoplasmosis is also noted to occur in immunocompetent adults [2-3]. The reason behind this occurrence is not well established, but some studies have attributed it to abnormal T cell immunity [4].

Pathology of histoplasmosis

Once inhaled, *H. capsulatum* follows an incubation period of 7-21 days to transform into a pathogenic yeast phase. This form replicates within macrophages that carry the yeast from the lungs to virtually any organ [5-6]. Induction of adaptive immunity, particularly the Th1 response, is required for the activation of macrophages and efficient clearance of the yeast. The development of severe clinical disease in immunocompetent adults is dependent on the large inoculum size of the fungus or ineffective clearance of the fungus [7].

The strains of *H. capsulatum* are unique and can alter the body’s immune system. The fungus is capable of evading intracellular killing by phagocytes, with mechanisms to degrade reactive oxygen species, regulate lysosomal pH, and capture essential nutrients that might otherwise be deprived [8]. In addition, there is evidence that strains of *H. capsulatum* can suppress the production of proinflammatory TNF alpha from phagocytes and escape the defense mechanism of the body [9]. In both human and mouse infections, macrophages provide a niche for *H. capsulatum* proliferation that cannot be halted without healthy adaptive immunity. More specifically, proper defense against *H. capsulatum* requires the polarization of the CD4+ T cells, production of protective Th1-associated cytokines, suppression of detrimental Th2-and regulatory T cell (Treg)-associated cytokines, and generation of Th17 response. TNF alpha is an essential cytokine and plays an important role in the defense against *H. capsulatum* by suppressing Th2 and Treg numbers [7].

Histoplasmosis in RA

The major risk factors for disseminated histoplasmosis are living in endemic areas, age of more than 54 years, HIV infection, solid organ transplant, malignancies, use of TNF inhibitors, and use of corticosteroids equivalent to prednisone of more than 20 mg per day for more than three months. Most cases of disseminated histoplasmosis caused by the use of TNF inhibitors are reported in RA patients after sustained use for at least 15 months. Moreover, in vitro studies on TNF alpha inhibitors have shown the inhibition of lymphocyte and macrophage proliferation, causing increased growth of *H. capsulatum* [10]. However, the incidence of disseminated histoplasmosis in RA also extends to patients who are not on TNF alpha inhibitors [11]. Another interesting point noted from this study involving data analysis in RA and disseminated histoplasmosis is that the average daily dose of prednisone reported in these subjects was lower than 20 mg [11]. Various clinical epidemiological studies have indicated that patients with RA are more prone to opportunistic fungal infections than patients with other autoimmune diseases [12]. Similar supporting findings have been observed from the medical records in the National Institutes of Health-funded Rochester Epidemiology Project of individuals with RA [13].

RA is an autoimmune disease with a complex pathology that targets all lines of defense in the human immune system. Most importantly, the disease is associated with an abnormal T cell function and TNF response [14-15]. The adaptive immunity in RA is impaired by a constricted T cell receptor repertoire, which is crucial for naive T lymphocytes to recognize all potential harmful antigens [16]. In addition, the capacity of clonal expansion of naive T cells in response to a previously unknown antigen and the frequency of migration of newly generated naive T cells from the thymus into the periphery can be significantly reduced in RA [17]. The other hypothesis explaining an increased incidence of infections in RA is gene polymorphism in the TRAF1/C5 locus [18].

In histoplasmosis, immunoglobulin A molecules are the first immunoglobulins to act at the line of defense [19]. The impact of RA in causing abnormal glycosylation of immunoglobulins increases the risk of mucosal infections, including oral and pulmonary diseases [20].

Our patient had severe RA and had been on prednisone 10 mg twice a day for a duration of one month when he presented with respiratory distress. The gross examination of the oral mass, the radiological appearance of the lung, and the presence of a liver nodule, especially given his history of “chewing tobacco,” led to a high suspicion of malignancy. The malignancy was ruled out effectively by a tissue biopsy, and a diagnosis of disseminated histoplasmosis was established. The development of disseminated histoplasmosis is a common finding in patients receiving TNF inhibitors or high-dose corticosteroids for more than three months, but since our patient was not on any such treatment, the presence of this disease was unexpected. In our case, the presence of RA that can increase the risk of infection by causing defective cellular immunity was the most likely factor responsible for the disease.

## Conclusions

Our case had several interesting manifestations of disseminated histoplasmosis, such as an oral mass, pulmonary infiltrate, liver mass, and brain lesions in the absence of anti-TNF medication use. This case represents an ideal model to study the complex pathology behind the RA and disseminated histoplasmosis in adults living in areas endemic for *H. capsulatum*. The mortality from untreated disseminated histoplasmosis is near 100%, and the disease itself can progress rapidly, causing a fulminant decline within days to weeks. Current guidelines indicate screening of individuals living in areas endemic for *Histoplasma* only before initiating anti-TNF inhibitors. Our manuscript opens up a debate about the possibility of RA being an independent risk for opportunistic infections like disseminated histoplasmosis. The case report also highlights the effect of glucocorticoids on the adaptive immunity in RA and the development of opportunistic fungal infections even with a shorter duration of treatment (less than three months).

We conclude that it is advisable to keep a low threshold to evaluate this disease in symptomatic patients, regardless of the use of TNF inhibitors in RA. Specific experimental and epidemiological studies can be performed to examine the relationship between RA and similar indolent fungal infections.
